# A robot goes to rehab: a novel gamified system for long-term stroke rehabilitation using a socially assistive robot—methodology and usability testing

**DOI:** 10.1186/s12984-021-00915-2

**Published:** 2021-07-28

**Authors:** Ronit Feingold-Polak, Oren Barzel, Shelly Levy-Tzedek

**Affiliations:** 1 Department of Physical Therapy, Recanati School for Community Health Professions, Ben-Gurion University of the Negev, Beer-Sheva, Israel; 2 Sheba Medical Center, Ramat Gan, Israel; 3 Adi-Negev Rehabilitation Center, Nahalat Eran, Israel; 4 Sackler Faculty of Medicine, Tel Aviv University, Tel Aviv, Israel; 5 Ono Academic College, Kiryat Ono, Israel; 6 Zlotowski Center for Neuroscience, Ben-Gurion University of the Negev, Beer-Sheva, Israel; 7 Freiburg Institute for Advanced Studies (FRIAS), University of Freiburg, Freiburg, Germany

**Keywords:** Socially assistive robots, Stroke, Rehabilitation, Exergames, Participatory design, Co-design, In-the-wild-study, Trust

## Abstract

**Background:**

Socially assistive robots (SARs) have been proposed as a tool to help individuals who have had a stroke to perform their exercise during their rehabilitation process. Yet, to date, there are no data on the motivating benefit of SARs in a long-term interaction with post-stroke patients.

**Methods:**

Here, we describe a robot-based gamified exercise platform, which we developed for long-term post-stroke rehabilitation. The platform uses the humanoid robot Pepper, and also has a computer-based configuration (with no robot). It includes seven gamified sets of exercises, which are based on functional tasks from the everyday life of the patients. The platform gives the patients instructions, as well as feedback on their performance, and can track their performance over time. We performed a long-term patient-usability study, where 24 post-stroke patients were randomly allocated to exercise with this platform—either with the robot or the computer configuration—over a 5–7 week period, 3 times per week, for a total of 306 sessions.

**Results:**

The participants in both groups reported that this rehabilitation platform addressed their arm rehabilitation needs, and they expressed their desire to continue training with it even after the study ended. We found a trend for higher acceptance of the system by the participants in the robot group on all parameters; however, this difference was not significant. We found that system failures did not affect the long-term trust that users felt towards the system.

**Conclusions:**

We demonstrated the usability of using this platform for a long-term rehabilitation with post-stroke patients in a clinical setting. We found high levels of acceptance of both platform configurations by patients following this interaction, with higher ratings given to the SAR configuration. We show that it is not the mere use of technology that increases the motivation of the person to practice, but rather it is the appreciation of the technology’s effectiveness and its perceived contribution to the rehabilitation process. In addition, we provide a list of guidelines that can be used when designing and implementing other technological tools for rehabilitation.

*Trial registration:* This trial is registered in the NIH ClinicalTrials.gov database. Registration number NCT03651063, registration date 21.08.2018. https://clinicaltrials.gov/ct2/show/NCT03651063.

## Background

Retraining coordination of reach-to-grasp movements is one of the major functional goals of rehabilitation after stroke [1], as it is the basis of a substantial number of daily activities, such as reaching to pick up a cup for drinking [2]. Intensive, repetitive task-specific training [3–5], over multiple sessions, can improve arm function post stroke [1, 6]. However, intensive practice, which requires a large number of repetitions, is challenging both for the patient and for the therapist [4, 6], for a variety of reasons, including the limited time in the individual therapy sessions dedicated to both acquiring and practicing new abilities, the fatigue of the patient [7, 8] and the lack of motivation of the individual with stroke to keep on training alone [9]. Therefore, it is imperative to devise feasible, alternative methods for long-term rehabilitation [4], which do not depend solely on the availability of the therapist, to be used both in the rehabilitation center and in patients’ homes [10]. These methods need to promote and motivate patients to practice their exercise, in order to improve the function of the impaired arm [4]. In order for the patient to repeat a certain task many times, they have to be highly motivated and engaged [5]. One of the ways to enhance motivation and engagement is to gamify the task; in the context of rehabilitation, this would translate to gamifying the repetitive exercise. Gamification has been demonstrated to increase patient motivation, learning, confidence, and positivity through achievement and social interaction [11]. Competitive and cooperative gamified tasks have been shown to increase motivation and exercise intensity of stroke patients when playing with another patient [12] or when playing with a healthy individual as a partner [9]. These suggest that the presence of a partner and of competition increases motivation and engagement. This can be achieved, for example, using competitive elements (such as a score) on a computer screen, or by using interactive robots, which may take on the role of a competition partner, or a coach. Socially Assistive Robots (SARs) have been designed for this purpose [13–21].

Previous works [13–16], on short-term interactions with a SAR, suggest that incorporating SARs into a practice regime that calls for repetitive tasks can increase stroke patients’ motivation. In previous works, patients were asked to do tasks such as magazine stacking [13], button pressing [16] or to imitate movements made by the robot [13] while receiving feedback from a SAR in a one-session interaction. These foundational studies demonstrated the feasibility of such an interaction with stroke patients. However, it is not yet known whether stroke patients' motivation will be maintained during a long-term interaction with the SAR, and whether it can lead to an improvement in their functional ability—that is, their ability to perform everyday tasks with their impaired arm, such as reaching to pick up a cup and drink from it.

Our goals in the current work were therefore threefold: first, to build a platform for functional post-stroke rehabilitation, which can track the performance of patients over time. More specifically, we aimed to build two implementations of this platform, in which the instructions and the feedback to the patient are given either by a socially assistive robot or by a computer screen. Second, to conduct a usability study with stroke patients, who will undergo a long-term intervention in the clinic with these two implementations of the platform. Third, to measure the patients’ willingness to exercise with the platform following a long-term intervention with it (15 exercise sessions conducted over 5–7 weeks), and to test the differences in the willingness of participants to use the system when using the SAR configuration, compared to the computer one.

We hypothesized that participants in the SAR group would show greater motivation and willingness to keep on exercising with the system compared to the computer group as will be measured (i) by the usability questionnaire, and (ii) by the dropout rates.

## Methods

### Preliminary work

We developed the first pilot version of the platform, which we describe in detail below, and tested it in a series of studies with young and older healthy adults [18]. Our aims in that work were: (i) to test the effects of age on preferences when interacting with a SAR; (ii) to test the differences in the motivation of users to interact with a computer interface, compared to with a SAR; and (iii) to adapt the platform according to the input we received from healthy adults. Following these studies, we developed an advanced version of the platform, which included five exercise games (games 1,2,3,5,6 detailed in "The exercise sets"). Prior to introducing the platform to stroke patients, we conducted a focus-groups study with expert clinicians who work with stroke patients in their everyday practice [19, 20]. Based on their recommendations, which are detailed in [19, 20], prior to deploying it in a long-term intervention study with stroke patients, we made several changes to the platform, which are all listed in [19, 20]. For example: we added functional exercises which required the use of both arms (as opposed to a single arm), as one would be asked to perform in a standard rehabilitation session in the clinic; we added specific instructions on whether to use the impaired arm or both arms for each exercise; we added a demonstration video for each task, which can be shown if needed; we developed two modes of each exercise set, the first with unlimited time to complete the task, and the second with a time limitation, thus providing both a motor and a cognitive challenge. The guiding principles in designing the current platform follow the recommendations of the American Academy of Physical Medicine and Rehabilitation and the American Society of Neurorehabilitation, as presented by Winstein et al. [22]. Below, we describe the full platform for the first time in detail; we first give an overview of the system, then describe the exercise games and finally the different features and the technical details of the platform. Then, we report the between-subject results and the conclusions from a long-term usability study with 24 post—stroke participants, who were randomly allocated to two intervention groups: SAR and computer.

### System design rationale

#### Use of everyday objects

One of our main goals in the current study was the use of real objects from everyday tasks as part of the exercise gaming program. Hubbard et al. (2009) mentioned that when guiding application of task-specific training in clinical practice, a task-specific training should be repetitive and relevant to the patient and to the context [5]. Scharoun et al. and Rosenbaum et al. [23–27] demonstrated that our specific functional goal when reaching to an object (e.g., reaching for a cup in order to drink from it, or in order to pour water into it) affects the way we perform the movement. Reach-to-grasp movements often require precise application of grip forces, which relies on prediction and sensory feedback [28]. The impaired ability to regulate force application has an important effect on stroke patients’ independence level [29], as difficulties in applying and adapting adequate grip forces [30] limit their ability to perform daily activities [31].

Extensive research (e.g. [32] has been done in the past decade on the use of virtual reality (VR) technologies for rehabilitation, specifically of the upper-limb. While being relatively cheap, accessible and encouraging high-intensity training [33], one of the problems that still remains unsolved is that upper-limb kinematics when using VR have been reported to be altered compared to those of movements performed in physical environments [34, 35]. The virtual environment still lacks the ability to provide haptic feedback to the user [34] and thus does not enable practicing force regulation as part of reach-to-grasp training. Here, we use an engaging and motivating technology (SAR) to help users practice reach-to-grasp movements, in a physical environment, using real everyday objects, thereby enabling users to practice both the kinematics and the force regulation required in a reach-to-grasp task.

#### Personalization in rehabilitation

In the last few years, patient-centered care has been widely accepted as an essential component of healthcare and specifically in the rehabilitation care [36]. In previous works [15, 18–20, 37–39] one of the most repeatedly noted requirement of SAR for rehabilitation was the need for personalization of the system and of the interaction. The importance of tailoring the rehabilitation program for the individual needs of each person was also highlighted by Winstein and Varghese [40], who noted that researchers and clinicians should adopt a more patient-centered approach in clinical trial design and in clinical care. Rehabilitation, in its nature, is a long and dynamic process. Thus, in order for the robotic device to be effective for therapy and accepted by both the patient and the clinician, it has to be flexible and adjustable, taking into account the complexity of the specific impairment and the dynamic changes that the patient experiences. For the experience of the user to be positive, and to achieve engagement in the task, it is important to personalize the interaction according to the characteristics of the task, the age of the user, their needs, and to their changing and dynamic abilities. Below we describe the adapted characteristics of the current system to the individual user.

### The platform

#### Overview

**Fig. 1:**
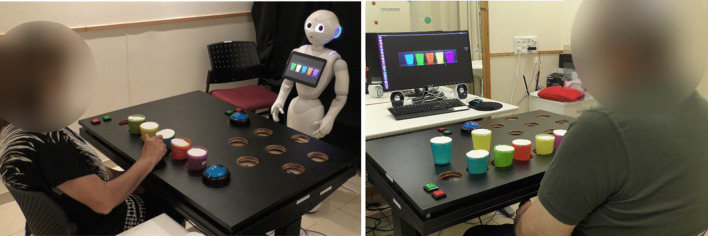
The two implementations of the stroke rehabilitation system. Left: the social robot Pepper gives instructions and feedback to the patient (ROBOT). Right: the instructions and feedback are presented on a computer screen (COMPUTER)

We developed a gamified platform for stroke upper-limb rehabilitation. The platform can be used in one of two configurations: in one configuration, the patient receives the game instructions and the feedback from a humanoid robot (the ROBOT configuration; we used the Pepper robot, Softbank Robotics Aldebaran), and in the other, from a standard computer screen (the COMPUTER configuration; see Fig. 1). Though the advantage of physical embodiment has been discussed previously [41, 42], this question has not been addressed in the context of a long-term interaction and more specifically, in the context of long-term rehabilitation. Our motivation for developing two configurations of the system is that most of homes and clinics have access to a computer rather than to a robot, and we thus aimed to test the added value of the SAR over the computer in terms of user motivation to engage in a long-term interaction in the context of post-stroke rehabilitation.

For the sake of brevity, we will refer to the ROBOT configuration when describing the platform, while the information is applicable for the configuration with the computer screen as well. That is, when information is described as presented on the robot’s tablet screen, it was presented on the computer screen in the COMPUTER configuration; when the robot gave audio instructions, the screen’s speakers emitted the exact same instructions in the COMPUTER configuration. Audio instructions were always accompanied by on-screen text with those same instructions: these were presented on the robot’s tablet screen in the ROBOT condition, and on the computer screen in the COMPUTER condition.

In each of the seven instrumented exercise sets which we developed, patients practice reach-grasp-and-place, or reach-grasp-and-manipulate movements, using real everyday objects such as cups, jars, keys, wallets, and drawers. These functional exercise sets enable the patients to practice both motor and cognitive abilities. In each exercise set, there are between four and seven levels of difficulty, with the level of difficulty being a function of: (i) the number of objects the participant manipulates during each trial (they start with a small number of objects and progress to manipulating more objects), (ii) the weight of these objects (they start with picking and placing lightweight objects and progress to heavier ones), and (iii) the height of the table or the shelf on which they have to place the objects (they start by manipulating objects at a standard table height (75 cm), or lower, and progress to shoulder height, as a function of their ability). Three of the exercise sets are designed to practice reach-grasp-and-place movements (exercise sets 1,2,5 detailed below), and the other four are designed to practice reach-grasp-and-manipulate (e.g., pick up a wallet and open its zipper to retrieve a key from it; exercise sets 3,4,6,7 detailed below). In each exercise set, the instructions to the participant indicate whether they should use only their impaired hand or both hands in order to complete the task. All the objects and the surfaces of the platform are equipped with tags and sensors, respectively, that enable tracking the objects’ end location, as detailed below.

We provide the details of the seven exercise sets below. Since they are all gamified, we refer to them as games.

#### The exercise sets (1–7)

*The Cup Game (1) and the Target Game (2)*

**Fig. 2:**
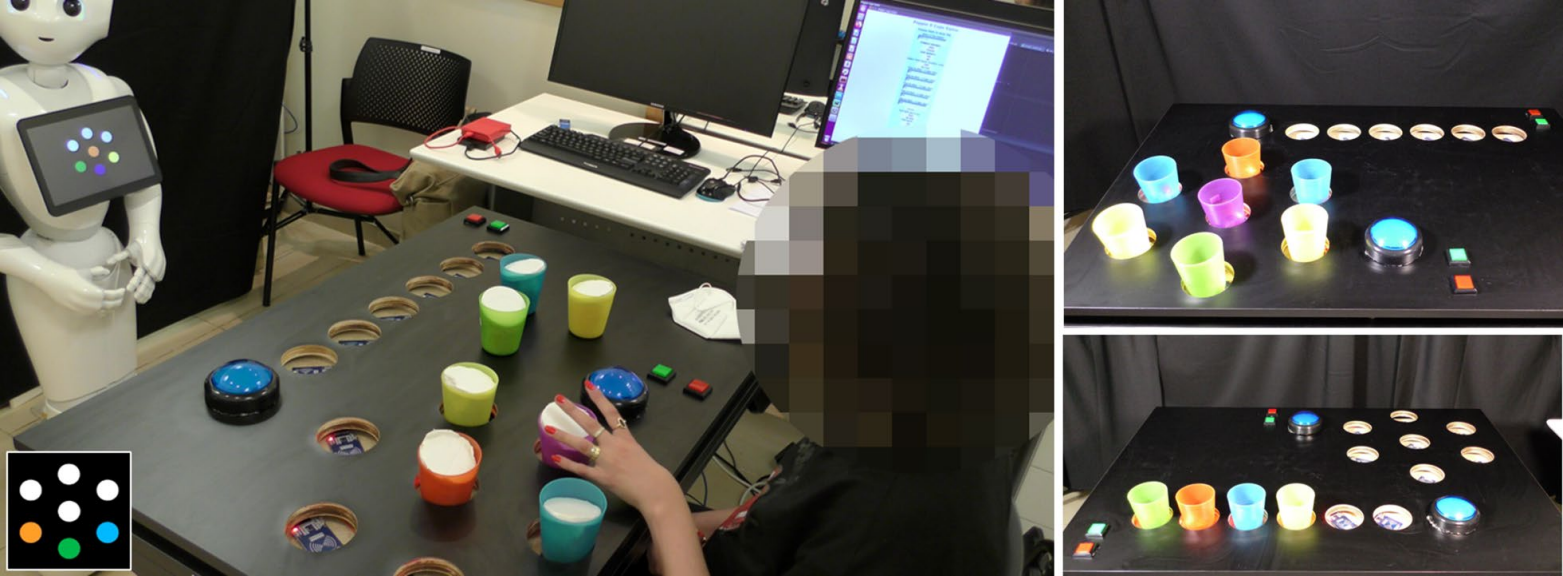
The *Target* and the *Cup Games*. Left: A patient playing the *Target Game* with the instructions provided by the robot. Inset: an example of instruction in this game, where three colored cups should be placed along a circle; white circles denote empty spaces. Top right: an illustration of the *Target Game* with all seven cup locations occupied. Bottom right: an illustration of the *Cup Game* with four occupied cup locations, out of the possible six

In each of twelve trials in the *Cup Game* [18], a row of colored cups is displayed on the robot’s tablet screen. The participant, seated next to an instrumented table, has to organize a corresponding set of colored cups on the table according to a picture shown on the robot's tablet screen (see Figs. 1, 2). There are four levels of game difficulty, depending on: (i) the number of cups, starting from three cups in the first (easiest) level, up to six cups in the fourth (hardest) one; and (ii) the weight of the cups: the cups can be either empty (34 g) or full (180 g) (for most patients, the empty cup is easier to lift, but for some the full cup provides stability); we used dry beans to fill the cups in the “heavy” configuration. The table on which this exercise set is performed is height adjustable, and was fit with a custom-built top plate, with 8-cm holes, so that the patients can comfortably place the cups in the designated locations, without the risk of knocking them over. This exercise set is considered the simplest one, since the spatial organization of the objects requires mostly a sideways movement of the arm, with little movement away from the body, or in the vertical dimension.

The *Target Game* is similar to the *Cup Game*, but rather than organizing the cups in a row, they are to be arranged in a circle, similar to a bullseye board, with six target locations arranged in a circle around a target location in the middle of the circle (see Fig. 2). In the *Target Game*, there are 21 trials with five levels of difficulty, starting from three cups in the first (easiest) level, and progressing to seven cups in the last (hardest) level. As in the *Cup Game*, the cups can be either empty or full. This game is more difficult than the *Cup Game* since it requires spatial perception and a multidimensional movement of the hand in different directions across the plane of the table (i.e., both sideways, and towards-and-away from the body).

*The Keys Game (3) and the Purse Game (4)*

**Fig. 3:**
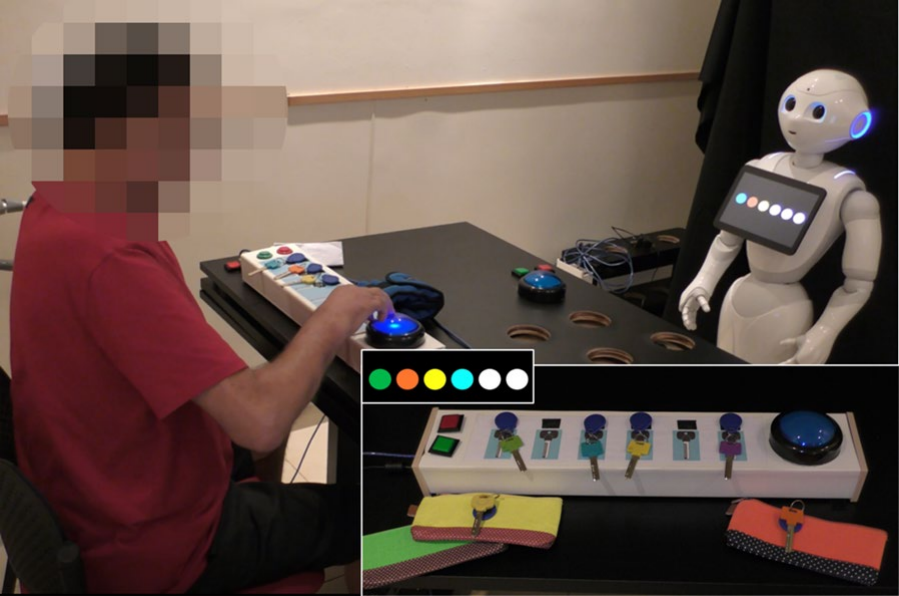
The *Keys* and the *Purse Games*. A patient playing the *Keys Game* with the instructions provided by the robot. Large inset: an illustration of the *Purse Game*, showing the keys, taken out of color-matched zipped purses, to be placed on the sensorized key hanger. Small inset: an example of instruction in either of these two games, where four colored keys should be placed on the key hanger; white circles denote empty spaces

In the *Keys Game*, the participant takes colored keys out of a box and places them on a key hanger according to an image displayed on the robot's tablet screen. The image on the robot's tablet screen is of a row of circles in different colors (see Fig. 3). In the box there are 12 keys, of different colors: the color of six of them matches the colors that participate in the game, and six other keys are used as distractors.

In the *Purse Game*, the participant takes the keys out of six different zipped purses, whose color matched that of the keys, and attaches each key to the key hanger according to the colors displayed on the robot's tablet screen (see Fig. 3). Both these exercise sets require performing a reaching movement, as well as manipulation of small objects.

*The Kitchen Game (5)*

**Fig. 4:**
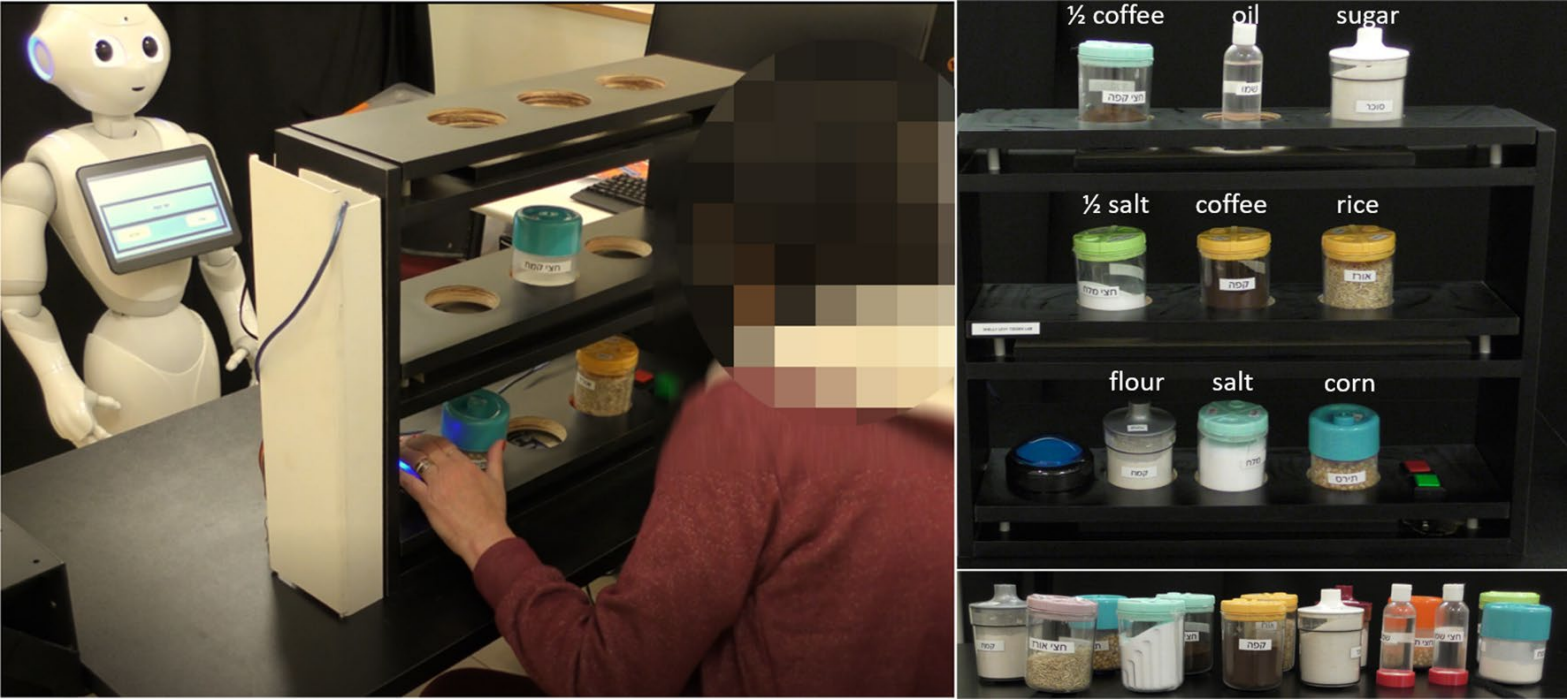
The *Kitchen Game*. Left: A patient playing the *Kitchen Game* with the instructions provided by the robot. Top right: an illustration of the *Kitchen Game*, with all nine shelf locations occupied. The white text above each jar/bottle indicates the content of each item, which is also printed on the item itself; “½” indicates that the jar or bottle is half full. Bottom right: The variety of kitchen items that are used in this exercise game

In the *Kitchen Game *[19, 20], in each of fifteen trials, the participant has to organize a set of labeled plastic jars on shelves at three different heights, according to an image shown on the robot's tablet screen (see Fig. 4). The jars are either half full or completely full with actual food items and condiments, such as salt, sugar, oil, coffee, corn kernels, etc., and a label indicates the contents of the see-through jar. There are seven levels of game difficulty. The difficulty is determined by a combination of: (i) the number of jars to be placed on the shelf; (ii) their weight (half or completely full), and (iii) the height of the shelf (there are 3 shelf heights). The first level has three lightweight jars placed on the lowest shelf (height of a standard table). In the seventh level there are nine jars, of different weights (110–400 g), placed on shelves at three different heights (see Fig. 4). The kitchen shelves were custom-built with 8-cm holes so that the patient can comfortably place the jars in the designated locations, without the risk of knocking them over. This game requires users to reach to different heights and lift different weights, as well as practice their spatial perception.

*The Black Jack Game (6)*

**Fig. 5:**
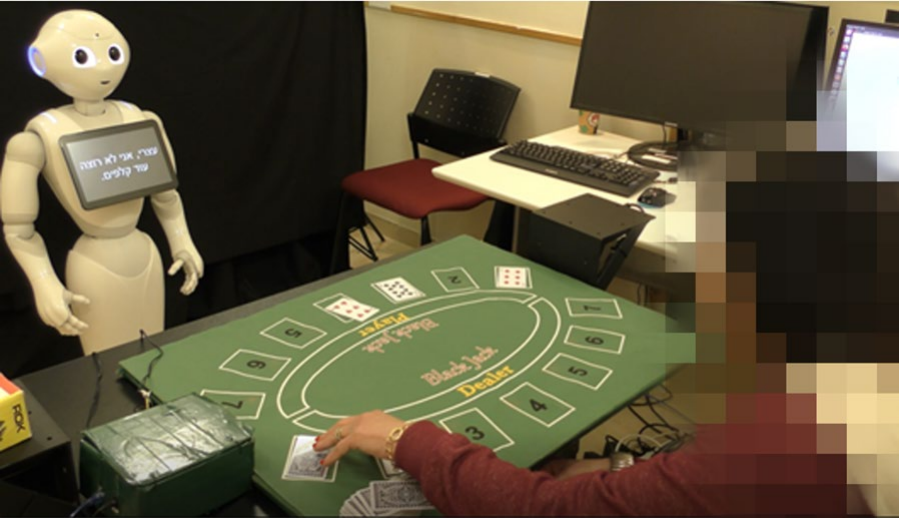
The Black Jack Game. A patient playing the Black Jack Game in the role of the dealer, with the robot in the role of the player. The cards, custom-printed on RFID tags, are placed on the sensorized table by the patient

In this implementation of the Black Jack card game, the participant is the dealer and the robot is the player (see Fig. 5). The rules of the game are presented by the robot at the beginning of the game. Then, the participant, in the role of the dealer, hands out the cards to the robot and to themselves, according to the rules of the game. A round is won when both the robot and the participant do not want to add more cards. The winner is the one for whom the sum of their card values is highest, but not higher than 21. We custom-printed a full deck of cards on Radio Frequency Identification (RFID) cards (see details in Sect. "Sensing apparatus" below), for this game.

*The Escape-Room Game (7)*

**Fig. 6:**
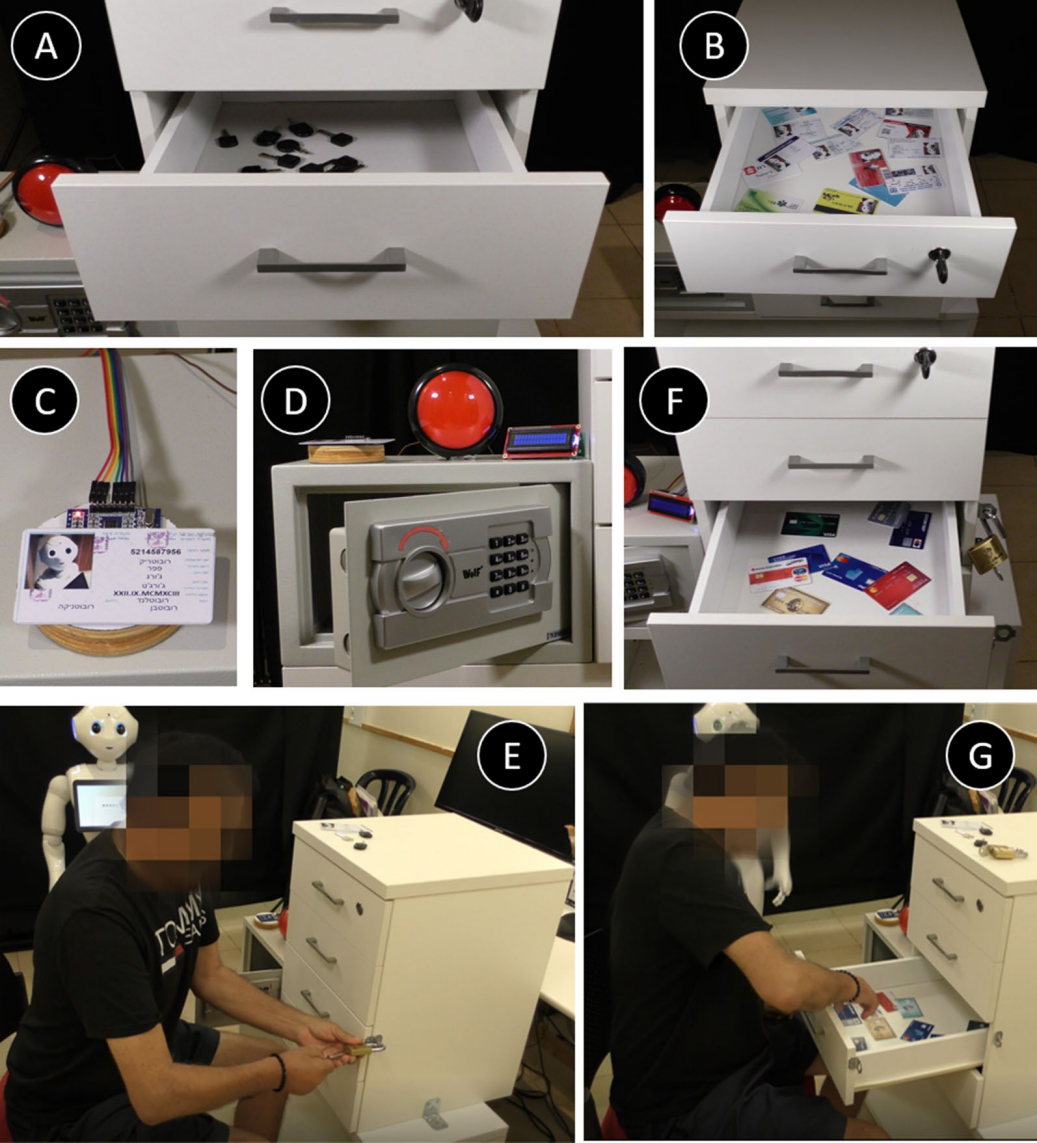
The Escape-Room Game. **A**–**E** some of the tasks of the exercise, including opening drawers to a specific distance, identifying a key or a card among distractors, punching numbers on a keypad, turning a lock, etc. **F**, **G** a patient performing the tasks in this exercise, corresponding to instructions 5 and 6 in the text

In this game, there is a background storyline, where the robot tells the participant it has to leave and go out on a drive (which usually provides some comic relief) and charges the participant with the mission of helping it find its ID card, keys, and its credit card. In a series of guided tasks (listed below) the participant is required to practice everyday actions such as opening a drawer by a gentle pull (to a specified distance), opening a lock using a key, pressing switches, etc., until the mission is completed. In the end, the participant is asked to organize all the objects they collected during this series of tasks into a wallet, thus practicing yet another everyday activity. In this exercise set, the participant practices manipulation of objects, bilateral movement of both hands, a controlled movement of the hand. Since the nature of this game was different than games 1–5 above, the progression in the game was not as described for the other games. When repeating this game more than once, participants could improve the time it took them to complete the task. The specific instructions and exercise game progression were as follows:

1. "The ID card you need is in the top drawer. The drawer is locked. The key to the top drawer is in the second drawer. Please open the second drawer only to the point where you see a red light turn on." A distance sensor installed in the back of the drawer chest was used to measure the extent to which the drawer was opened. The red light on the side of the drawer was lit only when the drawer was opened to exactly 30 cm. The goal of this task is to practice force regulation of the open motion of the drawer (see Fig. 6a)
2. "Look for the key of the top drawer in the second drawer." (The right key was placed among 20 other similar keys; see Fig. 6a)
3. "Use the key to unlock the top drawer and find the ID card in the top drawer." (The ID card was placed among 10 other cards; see Fig. 6b)
4. "Place the ID card on top of the card reader (see Fig. 6c) and use the last four digits of the ID card number as the code for the safe." (The participant must press keypads and turn the lock to complete this phase; see Fig. 6d)
5. "In the safe, there are keys for the lock of the third drawer. Find the right key and open the lock." (The right key was placed among 10 similar keys; see Fig. 6e)
6. "In the drawer, you will find my credit card." (The credit card was placed among 10 other cards; see Fig. 6f,g)
7. "Place the credit card on top of the card reader. Then open the fourth drawer until the last two digits of the card appear on the digital screen next to the drawer."
8. "Take the wallet out of the drawer and put all the items you found in it. "

While in previous work we described a preliminary version of the *Cup Game* [18], gave a brief description of the *Kitchen Game* [19, 20], and of the basic principles of the set of exercises [18–20], and the semi-autonomous characteristics of the system [20], in the current work, it is the first time we describe in detail all seven exercise sets, the different features of the system and its technical characteristics, alongside user reports from 20 individuals with stroke, who trained with the system for 15 sessions each.

#### Timing of the exercise sets

Each of the five exercise sets numbered (1–5) above can be played with no time limit to complete each trial of the task, or with a limited time to complete it. In the first configuration (unlimited time), the target image is displayed on the screen until the participant indicates they finished arranging the objects. The time a participant took to complete the task from start to end, as well as the average time it took the individual to complete the trials at each difficulty level of the task, are automatically recorded, and the clinician who is running the experiment is informed—via the user interface on the computer used to run the experiment. In the second configuration (limited time), the image with the target order of the objects disappears from the screen after an individually set time, according to the participant’s timing in the unlimited-time configuration. The time limit that is set is 2–4 s less than the time it took the user during the unlimited-time phase.

#### Verbal and visual instructions and feedback

After each trial, the robot either gives the participant feedback on the timing (e.g., “Try to do it faster next time”) or on their performance on the exercise set (e.g., “You succeeded!”, "You were not right, but try again"). The robot provides feedback by a combination of verbal response and visual feedback, which is displayed on the robot’s tablet screen. The robot’s responses are further accompanied by head and arm gestures (e.g., nodding, clapping, or dancing a victory dance; these gestures are not present in the COMPUTER condition). Importantly, these features of the embodied robot constituted the only difference between the intervention administered to the two groups. The robot is semi-autonomous in its function (as detailed below in "System autonomy"), such that after the clinician has set all the parameters of the game, the patient can exercise without the intervention of a clinician or a caregiver in the game. When the participant is wrong, an image with the correct order of the objects is displayed on the robot’s tablet screen, so the patient can see where they went wrong. In each trial, the patient collects points for the objects they ordered correctly. Since the Pepper robot does not support the Hebrew language, we could not use its built-in language capabilities. We, therefore, pre-recorded all the instructions and verbal feedback, using a female human voice. The same recordings were used for the ROBOT and for the COMPUTER configurations.

#### Exercise-set progression

In the very first ROBOT session, out of a total of 15 sessions, Pepper presents itself to the participant. In each of the following 14 sessions Pepper welcomes the participants with sentences such as "I'm so happy to see you again", "It is so nice that you came back" etc. In the COMPUTER configuration, there are no welcoming sentences, and the session starts by presenting the exercise game’s instructions. In each of the above-listed exercise games numbered (1–5), in order to indicate that they completed a task, the participants press a big push-button located on the right side of the table (see, for example, Fig. 3). In addition, after several trials at a certain level, the system offers the participant to advance to a higher level. In order to indicate their choice, the participant presses a green button to continue to the next level or a red button to stay at the same level. The red and green push buttons are placed on the side of the game table. Once pressing the buttons, the game continues automatically according to the participant's choice.

##### Pausing or stopping the game

In all seven exercise games, the participant can stop at any time if needed. Twice during the games numbered (1–5), after every two levels of the game, the robot offers the participant to rest and to join it for a stretching session. In addition, twice during each of these exercise games, after completing the third and the fifth levels, the system offers the participant to stop the game if desired. Furthermore, the participant can take a break or stop the game at any time, and when they continue playing, the game will resume from the same point at which it was stopped. The red and green push buttons, which indicate progression in the levels of the game, are also used to indicate whether the patient wants to continue playing or to stop playing when asked. In case of a fault of the system, it can be restarted from the same place where it stopped and the robot says "So, where were we? Let's continue from the same place".

#### Physical setup

##### Table height

For six of the seven exercise games we designed (games 1–6 listed above), we used a height-adjustable table. It is used to adjust the height of the table, depending on the height of the participant, and on the extent of their arm impairment. When the arm is weak (Fugl-Meyer Upper Extremity Assessment (FMA) score < 25/60 [43]), see below in "Usability study with post-stroke users)" we lower the table to the height of the patient’s thighs when seated; this helps them reach the objects on the table, a task which is harder for them when the table is higher [44, 45].

##### The exercise platform

On top of the height-adjustable table, we fixed a second top board, into which we drilled 8-cm holes, such that patients can comfortably place the objects in the designated locations, without the risk of knocking them over. These were used for the *Cup Game* and for the *Target Game* (Fig. 2). In the *Kitchen Game*, on top of each shelf, we fixed a second top board, into which we drilled three 8-cm holes as well, where the participant placed the jars and bottles (Fig. 4).

##### Sensing apparatus

In order for the system to detect where the objects are placed on the platform, and to detect completion of the task, we used an Arduino Mega board together with RFID readers. The RFID readers automatically identify and track tags that were attached to the different objects of the games (e.g., the keychains in Fig. 3). We placed RFID readers in all locations where users can place objects (the table, the kitchen shelves, the key hanger, and the Black Jack board). For the *Black Jack Game*, we used RFID cards, upon which we custom-printed the images of actual playing cards. In the *Cup Game*, *Keys Game,* and *Wallet Game*, the Arduino board is required to read 6 RFIDs at a time. In the *Target Game,* it is required to read 7 RFIDs in parallel, in the *Kitchen Game* 9 RFIDs are read in parallel, and in the *Black Jack Game* 14 RFIDs are read all at once. In the *Escape Room Game*, we used distance RFID readers, which indicated when the safe and the drawers were open and to what extent. The data are transported from the Arduino board, via a USB cable, to the computer that controls the game. In exercise games (1–5), when the big push button is pressed, the data are processed by the Arduino and then transported from the Arduino to the computer via a USB cable. In the *Black Jack* and *Escape Room* games, the Arduino board continuously read the RFIDs and transmitted the data to the computer. It takes approximately four seconds from the time the participant presses the push button until they receive feedback from the robot.

#### System autonomy

The gamified system was designed to be semi-autonomous [46], such that there is involvement of a third party in setting up the personalized exercise games for each participant, after which the patient can engage in a full exercise game without the intervention or the presence of a clinician or a caregiver. Before the game starts, the clinician inputs the following data into the computer, which controls the interaction: the patient's anonymized code, gender (so that the audio instructions will address the user using correctly gendered pronouns and verbs when speaking to the patient in Hebrew), whether it is their first interaction with the system, whether this is the first time they play this specific exercise game (*Cup, Target*, etc.), and whether the patient needs to watch a video of the instructions (the first time they play the game, or if they ask to watch it again). Setting up an exercise game may take up to seven minutes. It is common in the rehabilitation field that the patient needs assistance in the personalized setting of a device, after which they can train on their own. Then, the game instructions and feedback on performance are all provided by the robot or the computer screen, and the patient interacts only with the robot or the computer screen throughout the session (using the sensing apparatus as described in "Sensing apparatus"), with no involvement of a clinician or a caregiver. The system is a non-contact system, meaning there is no physical interaction between the robot and the participant.

### Usability study with post-stroke users

We conducted a long-term patient study in the ambulatory unit of the "Adi Negev" Rehabilitation Center in Israel. The research was approved by the institutional Helsinki ethical committee for clinical trials (SMC-5273-2018). All patients gave their written informed consent after they received a detailed explanation on the study from a medical doctor specializing in physical medicine and rehabilitation.

#### Participants

Twenty-four post-stroke patients (ROBOT group: 5 females, 6 males; age range 30–76 years, mean 54.8 ± 13; 52–245 days from stroke onset, mean 107 ± 54; FMA score 17–53/60, mean 42 ± 11. COMPUTER group: 6 females, 7 males; age range 40–77 years, mean 62.6 ± 15; 42–210 days from stroke onset, mean 111 ± 42; FMA score 17–54/60, mean 41 ± 12) participated in this study. Patients who met the following inclusion criteria were recruited to the study: (1) first unilateral stroke, confirmed by imaging; (2) Mini-Mental State Examination (MMSE) score ≥ 24/30 (for participants ≥ 65 years) [47] or the equivalent Montreal Cognitive Assessment (MoCA) score ≥ 20/30 (for participants < 65 years) [48]; (3) FMA [43, 49] score ≥ 16/60. The FMA assesses motor impairment of the paretic upper limb, where a higher score indicates less upper-limb impairment, and a score below 16/60 indicates the patient does not have the capacity to reach and grasp objects; (4) no other neurological and/or orthopedic condition that could affect arm movement. Exclusion criteria were as follows: (1) vision/hearing loss that limits the participant’s understanding of instructions, or; (2) aphasia that limits their understanding of instructions. 95% of the participants were right-handed. For 71% of the participants, the affected arm was their dominant arm.

#### Procedure

Participants were randomly allocated to perform a long-term functional exercise intervention in one of two groups ROBOT or COMPUTER, where instructions and feedback were provided by either the Pepper robot or a standard computer screen, respectively. The clinical outcome measures and the results of a third experimental control group, which did not undergo the intervention, are not within the scope of this paper and are not reported here. The allocation of participants to either of the intervention groups was randomized, and they were not familiar with the details of the intervention protocol in the other group.

Participation in the study was in addition to the conventional therapy the participants received as part of their rehabilitation program, as this system is designed to support the rehabilitation process and to provide additional training, on top of existing treatment. Following an initial assessment session, patients came to the facility two to three times per week over a period of 5–7 weeks, for a total of 15 therapy sessions with either the Pepper robot or the computer screen. The number of sessions was determined based on previous works [50, 51] that described significant clinical change in the arm and hand function of stroke patients following 12–15 sessions, at a frequency of three times per week. Each session lasted between 30 and 50 min, depending on participant's ability, fatigue, etc. In each session, the participant played one of the seven exercise games described above. Therefore, each participant played each of the seven exercise games in two of the sessions, and in the last (15th) session they were offered the option to choose which exercise game they wish to play for the third time. The patients had the option of playing more than one repetition of the same game in a single session if they so desired. After setting up the game parameters (e.g., whether this is the first session, whether a demonstration video is needed, etc.), a clinician was present in the room for safety reasons only and to solve any technical problems which may occur. There was minimal interaction of the participant with the clinician who was running the experiment. The participant was seated facing either Pepper or the computer screen (see Fig. 1), with their back to the clinician, who stood in the corner of the room, and did not intervene in the interaction with Pepper, with the computer, or in the sequence of the game. The level of game difficulty was determined at first according to the impairment level that was identified by the clinical tests, upon admission to the study. Then, after 4–6 trials in each game-difficulty level (e.g., with 3 cups in the *Cup Game*), the system asked the participant whether they wanted to progress to the next level of difficulty (e.g., with 4 cups in the *Cup Game*), or continue to exercise at the current level of difficulty.

#### Technical problems

During the experiment, we faced several technical problems. The RFID readers occasionally failed to correctly read the tags, and the system would indicate to the user that they were wrong, though they were, in fact, right. When this error was identified as systematic (the same RFID tag was consistently misread), the tag was immediately replaced by the clinician who was running the experiment and the patient could continue training. However, it also happened that a tag would on occasion be misread, while most of the time it was read correctly, with the pattern apparently random. If more than two mistakes occurred consecutively, the exercise game was restarted, which usually solved the problem. Rarely, a technical problem that required intervention of an engineer occurred (for example, when wires broke or when re-soldering of the wires was needed). Since there was no continuous engineering support at the clinic where the experiment was conducted when such a problem occurred, the exercise game was replaced by another one, and the participant could return to exercising with the interrupted exercise game on their next session, or when the problem was solved.

#### Outcome measures

##### User satisfaction evaluation questionnaire

Upon completing the full 15-session intervention program, each participant completed a custom-made user-acceptance questionnaire, in order to evaluate their acceptance of the novel system. The custom-made survey was based on the User Satisfaction Evaluation Questionnaire (USEQ). The USEQ is a six-questions questionnaire developed by Kizony et al. (2006) in order to assess virtual reality systems for rehabilitation [52, 53]. Participants are asked to rate their experience on a 1–5 Likert scale, where 1 indicates 'not at all'; and 5 indicates 'very much'. We added to the USEQ one question: "Would you like to keep using the system during your rehabilitation?" (see Table 1).

**Table 1:** User satisfaction evaluation questionnaire (USEQ)

Question	Response Not at all-very much
Q1. Did you enjoy using the system?	1 2 3 4 5
Q2. Were you successful using the system?	1 2 3 4 5
Q3. Were you able to control the system?	1 2 3 4 5
Q4. Was the information provided by the system clear to you?	1 2 3 4 5
Q5. Did you feel discomfort during your experience with the system?	1 2 3 4 5
Q6. Do you think that this system will be helpful for your upper-limb rehabilitation?	1 2 3 4 5
Q7. Would you like to keep using the system during your rehabilitation?	1 2 3 4 5

The questions from the user satisfaction evaluation questionnaire, where participants evaluate their experience with the system on a Likert scale of 1—"not at all" to 5—"very much"

##### Open-ended questionnaire

In addition to the USEQ, we asked the participants open-ended questions to allow them to describe their opinion on the system in detail. The open-ended questions are listed in Table 2.

**Table 2:** Custom-made open-ended questionnaire

What did you think of the system?
What did you like?
What did you not like?
What was your favorite exercise game? Why?
Was there an exercise game you did not like? What game? Why?
What would you add or change?

The custom-made open-ended questionnaire, where participants could freely evaluate and describe their experience with the system

##### Custom-made questionnaire for evaluation of technical limitations

Due to the technical problems which occurred during the experiment (as described above) we added three 1–5 Likert scale questions regarding how the system's mistakes influenced the willingness of participants to keep training with the system. These questions are detailed in Table 3.

**Table 3:** Participants’ evaluation of the effects of the system’s mistakes on their overall experience

Question	Response Not at all-very much
Q1. Did you feel the system was trustworthy?	1 2 3 4 5
Q2. To what extent did the mistakes of the system influence your trust in it?	1 2 3 4 5
Q3. To what extent did mistakes of the system influence your willingness to keep training with the system?	1 2 3 4 5

Participants evaluation of technical constraints, where participants evaluated their trust in the system on a Likert scale of 1—"not at all" to 5—"very much"

#### Data analysis

Data were analyzed using SPSS (Statistical Packages for Social Sciences, 26.0). We used the Mann–Whitney U test to analyze the different domains of the USEQ and of the custom-made questionnaire across the two intervention groups (ROBOT vs. COMPUTER), significance levels were set at p < 0.05. The answers of participants to the open-ended questions were analyzed per question and reported here as a percent of respondents who held a certain perspective, along with anecdotal direct quotes. We performed a Spearman rho correlation test in order to investigate the correlation between the impairment level, as defined by the FMA score at the entrance to the study, and participants’ evaluation of the contribution of the system to their upper-limb rehabilitation (question 6 on the USEQ) and their willingness to keep training with the system (question 7 on the USEQ). In addition, we analyzed the correlation between participants’ evaluation of the contribution of the system to their upper-limb rehabilitation and their willingness to keep training with the system.

## Results

### Participants

A total of 24 participants were randomly allocated into either the ROBOT (11 participants) or the COMPUTER (13 participants) intervention groups. The ambulatory rehabilitation unit was closed-down from mid-March 2020 to mid-May 2020 due to COVID-19. At that time, four participants were in the midst of the intervention. These participants could therefore not continue participating in the intervention program and were excluded from the study. One male participant in the ROBOT group was excluded due to this reason following four intervention sessions. Five participants in the COMPUTER group did not complete the intervention. Three of them were excluded due to the COVID-19 lockdown, which interrupted the intervention protocol. Of those, one was a male who had already completed seven intervention sessions by that point, and two were females who already completed four and nine intervention sessions. In addition, two women dropped out from the COMPUTER group since they did not wish to continue the intervention (20% of the remaining 10 participants): a 40-year old woman following four intervention sessions, and a 59-year old woman following eight sessions. None of the participants in the ROBOT group dropped out of their own will.

Ten participants in the ROBOT group and eight participants in the COMPUTER group completed 15 intervention sessions. Overall, participants took part in a total of 306 sessions altogether (154 sessions for the ROBOT group, 152 sessions for the COMPUTER group).

### Results from the modified user satisfaction evaluation questionnaire (USEQ)

**Fig. 7:**
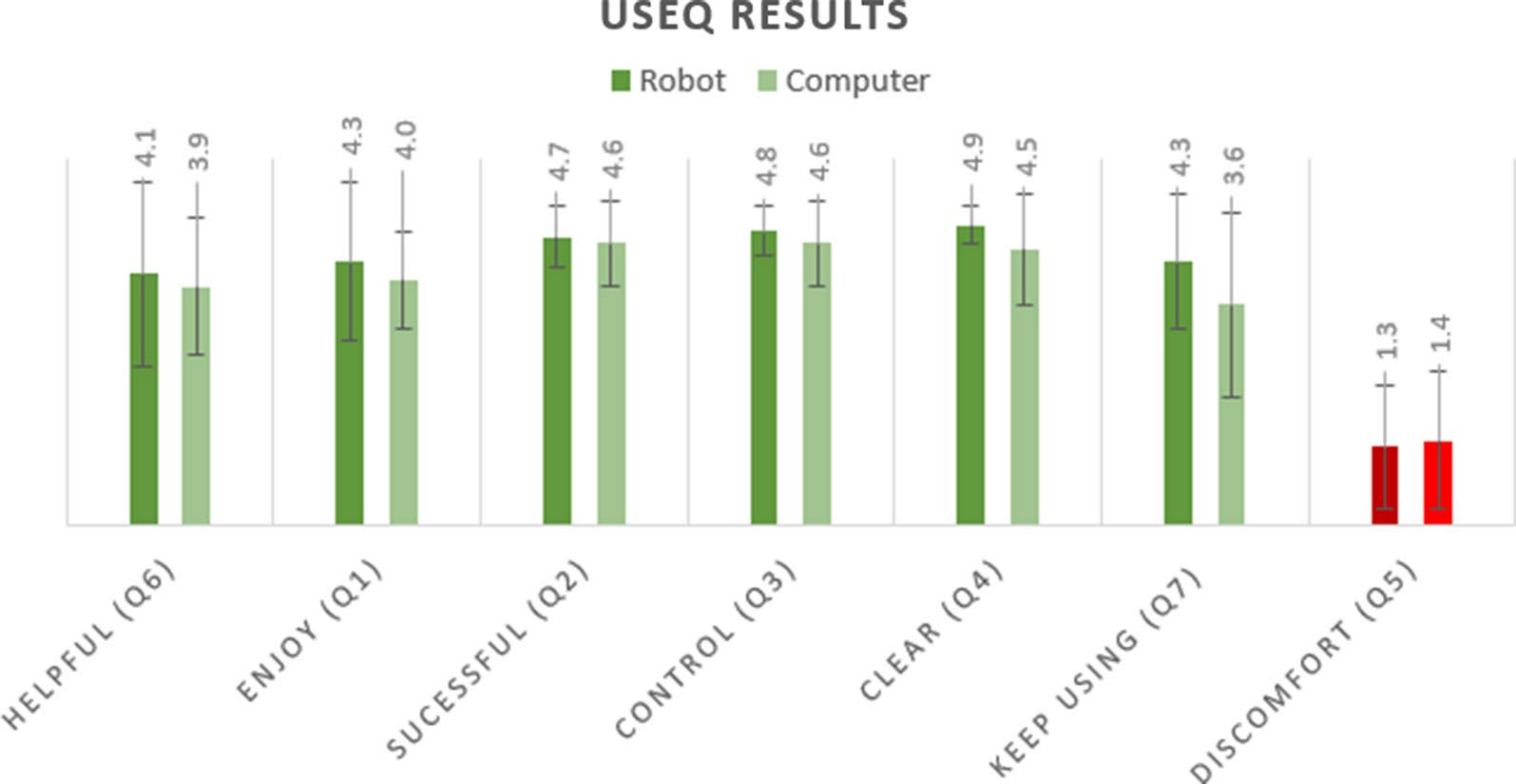
Results of the user satisfaction evaluation questionnaire (USEQ), mean ± SD. A score of 1 denotes "not at all" and 5 denotes "very much". The green bars denote the responses to the questions for which a higher rating indicates a more positive experience with the platform, and the red bars denote the responses to the question for which a lower rating indicates a more positive experience with the platform

All results from the USEQ are summarized in Fig. 7. The mean score for the question, "Do you think that this system was helpful for your upper-limb rehabilitation?" was 4.1 ± 1.5 out of 5 for the ROBOT group and 3.9 ± 1.1 for the COMPUTER group (Z = − 0.717, *p* = 0.473). To the question, "Did you enjoy using the system?" participants in the ROBOT group gave an average score of 4.3 ± 1.3, and in the COMPUTER group, it was 4.0 ± 0.8 (Z = − 1.346, *p* = 0.178). When asked "Would you like to keep using the system during your rehabilitation?" participants in the ROBOT group responded with an average score of 4.3 ± 1 and in the COMPUTER group the average score was 3.6 ± 1.5 (Z = − 1.052, *p* = 0.293). When comparing the experience of both groups, even though the ratings for the ROBOT group were higher for all parameters, there was no statistically significant difference between the groups in their rating for any of the USEQ questions. We found no correlation between the initial impairment level, as defined by the FMA, and either the participants’ evaluation of the contribution of the system to their upper-limb (*r*_s_ = − 0.007, *p* = 0.978) or to their willingness to keep training with the system (*r*_s_ = 0.161, *p* = 0.536). We found a strong correlation between participants' evaluation of the contribution of the system to their rehabilitation and their willingness to keep training with it (*r*_s_ = 0.774, *p* < 0.0001).

### Results from the open-ended questionnaire

There was no clear agreement among participants on what aspects of the exercise program they preferred. Fourteen out of 18 (78%) mentioned they enjoyed the motor aspect of the task, and 11 out of 18 (61%) mentioned they enjoyed the addition of a cognitive challenge (memory and spatial perception). Nine out of 18 participants (50%) noted that their favorite exercise game was the *Escape Room*, as it included both motor tasks and a thinking challenge; participants noted it was like solving a riddle. Two participants noted that it was too short in their opinion and that they would have liked more tasks to be added to it. Eight participants (44%) mentioned they liked all the exercise games.

Thirteen out of 18 (72%) of the participants mentioned they liked the diversity of the games and the addition of the dual-task cognitive challenge. Three participants (17%) noted they preferred the reach-grasp-and-place task games (games 1, 2, and 5), and three participants noted they preferred the reach-grasp-and-manipulate games, which required manipulation of objects (games 3, 4, 6, and 7). Importantly, five participants (28%) noted they would like to have a greater diversity of exercise games so that in each session there would be a new kind of game (in the current setup, with seven games used for 15 sessions, there was a repetition of games across sessions). In addition, six participants (33%) mentioned they would enjoy a more challenging task in terms of both motor control (for example, heavier weight, more levels of the game) and the cognitive demand (for example, more riddles). Finally, four participants (22%) mentioned they would rather have a system that can automatically detect their motor and cognitive abilities as well as their performance, and personally adjusts the level of the training and the feedback according to their motor and cognitive performance. Two participants from the ROBOT group mentioned they would like the robot to be dressed to make it more human-like. When participants were offered to choose what game to play on their last session, five out of 10 participants in the ROBOT group chose either the *Cup* or the *Target* games in the limited-time configuration; four participants chose each a different game; one participant did not choose a game. In the COMPUTER group, the distribution was as follows: two participants chose the *Target Game*; two—the *Escape Room*; two—*Black Jack*; one—the *Keys*; one—the *Kitchen Game*.

Below we quote several anecdotal statements made by the participants:

"The robot made me regain trust in my arm" (P07, ROBOT group);

"In none of the other therapy sessions I took part in (not as part of the experiment) was there a focus on the hand like here; here I dared to do things with the hand that I did not think I would succeed in doing, and it helped me believe I can do it at home as well" (P14, ROBOT group);

"I really liked the games, I felt you exactly identified the problem and hit the bullseye in treating it" (P13, ROBOT group);

"I only want to come to the sessions with the robot" (out of all the rehabilitation activities he was engaged in at the time) (P03, ROBOT group);

"I think that a bigger diversity of games is needed, it is boring to play the same game several times" (P14, COMPUTER group).

### Technical problems

There was no influence of the technical problems on participants’ acceptance of the system following the long-term intervention, and there was no difference in this regard between the two intervention groups. Participants in both groups reported they found the system trustworthy (ROBOT: 4.3 ± 1, COMPUTER: 3.9 ± 1.1; Z = − 0.910, *p* = 0.363). Participants reported that the mistakes of the system did not influence their trust in the system (ROBOT: 2.0 ± 1.2, COMPUTER: 2.0 ± 1; Z = − 0.188, *p* = 0.851) or their willingness to continue training with it (ROBOT: 1.6 ± 1.3, COMPUTER: 2.0 ± 1.4; Z = − 0.680, *p* = 0.496).

## Discussion

Here, we describe a robot-based gamified exercise system for long-term post-stroke rehabilitation, which can track the performance of patients over time. The system also has a computer-based configuration (with no robot) and includes a gamified set of functional tasks from the everyday life of the person, such as reaching to a cup or turning a key in a lock. We also report the results of a patient usability study, where 24 stroke patients were allocated into two intervention groups of the exercise program with these two platforms over a 5–7 week period, 2–3 times per week, for a total of 306 sessions.

The system we developed is novel in two main aspects: (i) the design and implementation of exercise games for stroke patients using reach-to-grasp movements to real physical objects; (ii) the usability testing of a long-term interaction of stroke patients with a humanoid robot and a computer screen for rehabilitation. Our study showed that patients found the gamified system engaging and motivating for rehabilitation following a long-term intervention. They found the system to address their needs in upper-limb rehabilitation and expressed their desire to continue training with the system even after the study ended. Participants in the ROBOT group rated the system higher on all parameters; however, as opposed to our first hypothesis, we found no statistically significant difference between the groups in their evaluation of the system, potentially due to the small sample size. Moreover, when examining the dropout rates, 20% of the participants in the COMPUTER group dropped out due to them being unwilling to continue the intervention, whereas none of the participants in the ROBOT group dropped out of their own will, which implies the motivating potential of the SAR over a computer, confirming our second hypothesis. There was a significant correlation between the appreciation of participants of the contribution of the system to their rehabilitation and the willingness of participants to keep training with it. Thus, it is not the mere use of technology that increases the motivation of the person to practice, but rather it is the appreciation of the technology’s effectiveness and contribution to the rehabilitation process.

As was stressed by Brackenridge et al. [3] in a review of robotic devices for rehabilitation, the goal of the system is to augment the work of the clinician, not replace it. It is designed to complement the one-on-one sessions with the clinician and to help the clinician and the patient achieve repetitive task-specific training in an engaging and motivating manner while using everyday tasks and objects.

### Long-term SAR study—lessons learned

To the best of our knowledge, this is the first study to evaluate a long-term intervention using a SAR with post-stroke patients in a rehabilitation center, as part of their conventional rehabilitation program. Though in the domains of health care and therapy there is great potential for social robots to assist users over extended periods of time [for examples see [54, 55]], there is still a limited number of works describing longitudinal studies within this domain [56]. Leite et al. (2013), in a survey of social robots for long-term interaction, noted several reasons for this. First, longitudinal studies are much more laborious and time-consuming than short-term studies, especially in ecological environments and in the wild. Second, only in the last few years technology has become robust enough to allow for some degree of autonomy when users interact with robots for extended periods of time [56]. In the current study, we placed the system in a rehabilitation center as part of patients' scheduled rehabilitation program, which we believe was a facilitator to the success of the implementation of the system. The described system was built and developed by our multidisciplinary lab team, which included both a physiotherapist who specialized in post-stroke rehabilitation and engineering students. We believe that a multidisciplinary team is a central component in the success of this platform. We faced several technical challenges, as described. Technical problems are part of any technology implementation, specifically of novel prototype devices. Therefore, having quick-responding technical support, and training the clinician to solve basic technical problems which may occur, is essential for the success of technology implementation in a rehabilitation setting. Future studies on SAR interaction should strive to use a room with a one-way mirror so that the participant will be able to interact safely with the system without the presence of research assistant, who will be sitting on the other side of the glass and will be able to see the participant and to intervene in case of a technical failure or if another assistance is required. When performing in-the wild studies, where the goal is to provide treatment to the patients, and, at the same time, to collect all relevant data, a balance between the two should struck. Performing too many clinical tests, or filling out too many questionnaires, could be exhausting to the patients and may interfere with their ability to benefit from the treatment; at the same time, not collecting the relevant data may interfere with the ability to reach a conclusion regarding the efficiency of the treatment, or the patients’ perception of the process. Therefore, our recommendation, when performing a longitudinal study, is to allow, in addition to the pre and post-tests, for five minutes at the start or end of each session to ask three key questions, to enable tracking a potential change over time, without exhausting the patients.

### Suggested guidelines for future designs of SARs for rehabilitation

Matarić et al. [15] suggested that the design of interactions with social robots for post-stroke rehabilitation should follow two guiding principles: (i) high intensity of task-specific training and (ii) a system that will be engaging and user-friendly. In the robot-based system that we developed, we followed these guidelines.

Based on our earlier work [18–20] and the current study we added several guidelines which expand these recommendations: (i) *Task variety:* For a system to be applicable to a wide variety of patients and different levels of impairments, and in order for it to engage patients in the long-term, there should be a variety of tasks, with different levels of complexity, which can be executed by both low-functioning and high-functioning patients. Users should be able to progress in the task according to their ability and motor performance. The participants in our study highlighted the variety of the tasks this platform offered to practice on, which they did not get the opportunity to practice in other therapy sessions they received as part of their standard rehabilitation program. (ii) *Communication:* The instructions given to the user should be simple, gradually increasing in difficulty, and spoken slowly and clearly. However, the response time of the robot should be as fast as in human–human interaction [18]. From our experience from the current study and from previous ones [18, 20], the response time of the system is longer than 4–5 s participants experience it as slow, which causes frustration. We added to the robot a reaction of "I'm checking" if it took it longer than four seconds to examine whether the order matched the displayed image, so the participant will not experience the robots' response time as too long (for more on the effect of timing on users’ perception of HRI, see [57]. (iii) *Fatigue management:* Since stroke patients experience frequent fatigue [7, 8] and muscle weakness, patients should have the ability to rest when needed. When the patient is fatigued and cannot complete the task without using undesirable compensatory movements [10], either the patient should rest, or the session should end. In the current system, in addition to offering the participant to rest or to pause when desired, we also added built-in stretching breaks. (iv) *Feedback and reward:* users need to receive feedback on their performance and on their results, as this is an essential component of their motor learning [58]. However, as the participants in our study noted, the feedback should be given in a manner and at a frequency that will not negatively affect their compliance to keep on training. Some of the participants in our study, especially the younger ones (< 45 yo) mentioned they do not wish to receive verbal feedback on their performance after each trial, but would rather receive verbal feedback after several trials and visual feedback (like the sign of raised thumb for "like") following the other trials. In addition, they mentioned they would like to receive feedback on their motor performance. That is, they sought feedback on their body movements as they performed the task, whether they involved any compensatory movements, in addition to their task performance. We are currently in the process of developing this capability [10]. (v) *Personalization of the system and of the interaction:* The value in adapting the rehabilitation program to the personal needs of the patient was also stressed by the participants in our study, who mentioned the importance of personalizing the design of HRI and human–computer interaction (HCI) and tailoring it to the specific task and patient needs. They mentioned they would like the system to be able to adapt to their personal performance, e.g. by adapting the feedback to their movement patterns, and by automatically progressing through the exercise game levels based on their success rates. Personalization is an essential component of trust establishment and an essential component when aiming to foster motivation.

### Trust and SAR in a long-term interaction

In previous works [59–61], the importance of trust in the context of SAR for rehabilitation has been discussed. Due to the limitations and subsequent frustration, a stroke patient may experience because of their impairments, a system that is designed for stroke patients should be as trustworthy as possible. Kellmeyer et al. (2018) stressed that this is especially important in vulnerable populations such as neurologically impaired patients, where interactions with robots are designed to assist them in maintaining a training regime, and where establishing long-term trust between the user and the robot is essential [60, 61].

In the current work we showed that—quite surprisingly—although there were faults of the system, in the long-term, this did not affect the evaluation of participants of the system as trustworthy or their willingness to keep training with it. As one of the participants noted: "humans also make mistakes". That is, in the long-term, the faults of the system did not affect the overall acceptance of the system by the participants. This could be due to their understanding that this was a prototype system and to the research team’s ability to solve the technical faults on the spot when possible.

### SAR for rehabilitation during the COVID 19 pandemic

In March 2020, the World Health Organization declared COVID-19 a pandemic. The rehabilitation world is now facing new challenges because of the requirement for social distancing, especially in at-risk populations. Khan and Amatya [62] noted the following two challenges that the realm of rehabilitation faces in light of COVID-19: (1) Providing safe physical environments within rehabilitation wards that comply with social distancing and hygiene; (2) mitigating risk (as able) for a potential COVID-19 exposure to patients and staff. The requirement to keep a social distance and reduce physical contact stresses the need for alternative rehabilitative tools, such as SARs, to enable patients to have an uninterrupted (even if modified) rehabilitation regime.

*Study limitations and recommendations for future studies:* While we achieved the aim of this study, i.e., testing the usability of the system among the actual stakeholders, post-stroke individuals, it would be essential to collect further data to investigate the clinical benefit of using SAR for long-term rehabilitation.

We attempted to strike a balance between positively worded questions (as in the USEQ) and negatively worded questions (as in the open-ended questionnaire we used); future studies need to maintain a balance between positively and negatively worded questions when studying the perceptions of users.

While there were no colorblind participants in the current experiment, it would be of value to verify in future work that the use of color as a distinguishing feature in the exercise games does not influence the ability of individuals with color blindness to benefit from these exercises.

While previous research (Lee et al. [63]) found an embodied robot to be preferred by participants over a virtual avatar, it could be instructive to explore, in future research, a modified version of the computer condition, which would include a welcome message and celebratory responses conveyed by, for example, an avatar.

We are interested in open-sourcing the tools from this project in the future. Interested researchers should contact the corresponding author.

## Conclusions

We demonstrated the feasibility of using the platform we developed for a long-term rehabilitation with stroke patients in a clinical setting and found a strong trend of acceptance of the SAR by post-stroke patients following this long-term interaction. The novelty effect of the SAR did not wear off following 15 intervention sessions. We showed that it is not the mere use of technology that increases the motivation of the person to practice, but rather it is the user’s appreciation of the technology’s effectiveness and contribution to the rehabilitation process. We found that system failures did not affect the long-term trust that users felt towards the system. In addition, we provide a list of guidelines that can be used when designing and implementing other technological tools for rehabilitation. Future research should examine the clinical contribution of SARs for the rehabilitation of persons with stroke, and incorporate feedback on movement quality.

## Data Availability

The datasets used and/or analyzed during the current study are available from the corresponding author on reasonable request.

## Abbreviations

RTG: Reach-to-grasp
SAR: Socially assistive robot
FMA: Fugl Meyer Assessment
MMSE: Mini Mental State Examination
MoCA: Montreal Cognitive Assessment
HRI: Human robotic interaction
HCI: Human computer interaction
USEQ: User satisfaction evaluation questionnaire
COVID-19: Coronavirus disease 2019
